# Relaxin Protects Rat Lungs from Ischemia-Reperfusion Injury via Inducible NO Synthase: Role of ERK-1/2, PI3K, and Forkhead Transcription Factor FKHRL1

**DOI:** 10.1371/journal.pone.0075592

**Published:** 2013-09-30

**Authors:** Konstantin Alexiou, Manuel Wilbring, Klaus Matschke, Thomas Dschietzig

**Affiliations:** 1 University Heart Center Dresden, Department of Cardiac Surgery, Dresden, Germany; 2 Immundiagnostik AG, Bensheim, Germany; 3 Charité-University of Medicine Berlin (Campus Mitte), Department of Cardiology and Angiology, Berlin, Germany; University of Pittsburgh, United States of America

## Abstract

**Introduction:**

Early allograft dysfunction following lung transplantation is mainly an ischemia/reperfusion (IR) injury. We showed that relaxin-2 (relaxin) exerts a protective effect in lung IR, attributable to decreases in endothelin-1 (ET-1) production, leukocyte recruitment, and free radical generation. Here, we summarize our investigations into relaxin’s signalling.

**Materials and Methods:**

Isolated rat lungs were perfused with vehicle or 5 nM relaxin (n = 6–10 each). Thereafter, experiments were conducted in the presence of relaxin plus vehicle, the protein kinase A inhibitors H-89 and KT-5720, the NO synthase (NOS) inhibitor L-NAME, the iNOS inhibitor 1400W, the nNOS inhibitor SMTC, the extracellular signal-regulated kinase-1/2 (ERK-1/2) inhibitor PD-98059, the phosphatidylinositol-3 kinase (PI3K) inhibitor wortmannin, the endothelin type-B (ETB) antagonist A-192621, or the glucocorticoid receptor (GR) antagonist RU-486. After 90 min ischemia and 90 min reperfusion we determined wet-to-dry (W/D) weight ratio, mean pulmonary arterial pressure (MPAP), vascular release of ET-1, neutrophil elastase (NE), myeloperoxidase (MPO), and malondialdehyde (MDA). Primary rat pulmonary vascular cells were similarly treated.

**Results:**

IR lungs displayed significantly elevated W/D ratios, MPAP, as well as ET-1, NE, MDA, and MPO. In the presence of relaxin, all of these parameters were markedly improved. This protective effect was completely abolished by L-NAME, 1400W, PD-98059, and wortmannin whereas neither PKA and nNOS inhibition nor ETB and GR antagonism were effective. Analysis of NOS gene expression and activity revealed that the relaxin-induced early and moderate iNOS stimulation is ERK-1/2-dependent and counter-balanced by PI3K. Relaxin-PI3K-related phosphorylation of a forkhead transcription factor, FKHRL1, paralleled this regulation. In pulmonary endothelial and smooth muscle cells, FKHRL1 was essential to relaxin-PI3K signalling towards iNOS.

**Conclusion:**

In this short-time experimental setting, relaxin protects against IR-induced lung injury via early and moderate iNOS induction, dependent on balanced ERK-1/2 and PI3K-FKHRL1 stimulation. These findings render relaxin a candidate drug for lung preservation.

## Introduction

Ischemia-reperfusion (IR) injury [1], which is characterized by non-cardiogenic pulmonary edema formation associated with an increase in pulmonary artery pressure and hypoxemia, is the major reason for early graft dysfunction in clinical lung transplantation. The incidence of early graft dysfunction has been estimated to be between 10 and 25%, with a severity ranging from very mild acute lung injury to ARDS, and it is clearly known that the quality of lung preservation is a key determinant herein. Early graft dysfunction represents the leading cause of early death after transplantation [1]. It is established that recruited inflammatory cells, accompanied by dysfunctional endothelial cells, produce a variety of mediators and enzymes resulting in the above-mentioned cascade of events [2]. A hallmark if pulmonary IR injury is the concomitant up-regulation of iNOS and diverse processes generating reactive oxygen and nitrogen species (ROS, RNS). In concert, this precipitates impaired bioavailability of NO and toxic effects of peroxynitrite [2].

We have recently provided experimental evidence that the peptide human relaxin-2 (relaxin), a member of the insulin superfamily, exerts a protective effect in IR-induced lung injury [3]. Relaxin caused a marked reduction of biochemical and morphological markers of pulmonary injury, particularly of the proteolytic enzyme, neutrophil elastase (NE, a mediator of alveolar destruction); the enzyme myeloperoxidase (MPO, a marker for neutrophil accumulation in tissues); and a reactive aldehyde, malonyldialdehyde (MDA, an end-product of peroxidation of cell membrane lipids caused by oxygen-derived free radicals). Relaxin down-regulated endothelin-1 (ET-1) secretion and decreased vascular permeability resulting in a significant reduction of pulmonary edema [3].

Here, we summarize our investigations into the signal transduction of this relaxin effect: The peptide is shown to exert its protective effect via early and moderate iNOS induction dependent on balanced ERK-1/2 and PI3K stimulation.

## Materials and Methods

### Isolated Perfused Lung

The study conforms to the European Commission Directive 2010/63/EU. According to German animal welfare regulations, the killing of laboratory animals for mere excision of organs, without prior experiments performed, does not pose an animal experiment and an approval is therefore not required. Male Wistar rats, weighing 300 to 350 g, were selected for this study. For excision of the lung lethal anesthesia with thiopental sodium (80 mg/kg body weight (BW) i. p.) was performed. After cessation of the corneal reflex, animals were exsanguinated by cutting the carotid artery and the jugular vein. A tracheotomy permitted positive pressure ventilation with a small animal respirator at 60 strokes/min, tidal volume 8–10 ml/kg BW, 1 mm Hg positive end-expiratory pressure, gas mixture 95% O_2_ and 5% CO_2_. A median sternotomy was performed, a cannula was placed into the pulmonary artery, and the heart was removed to allow passive drainage of the pulmonary effluent from the pulmonary veins. Perfusion was carried out with Krebs-Henseleit buffer containing composition in mmol/l: NaCl, 127; KCl, 3.7; CaCl_2_, 2.5; KH_2_PO_4_, 1.2; MgSO_4_, 1.1; NaHCO_3_, 25.0; glucose, 10; pyruvate, 1.8; and *N*-2-hydroxyethylpiperazine-*n*-2-ethanesulfonic acid (HEPES), 5.0; pH was 7.35–7.40 at 37.5 C. The lungs were placed in a humidified temperature-equilibrated organ chamber, freely suspended from a force transducer for continuous monitoring of weight. For the washout of blood, the first 75 ml of perfusate were discarded. In a recirculating system, the lungs were perfused at a constant flow of 0.03 ml/g BW. Only lungs were selected for the present study that showed a constant mean pulmonary mean arterial pressure (MPAP) of 6–8 mm Hg, zero-referenced at hilus, a constant peak inflation pressure of 7 to 10 mm Hg, no weight gain <50 mg/h, and no signs of haemorrhage, oedema or atelectasis during an initial steady-state period of at least 30 min.

### Experimental Protocol

Each lung was perfused with 40 ml of buffer in recirculatory mode, the entire perfusate was rapidly frozen in liquid nitrogen and stored at −70°C for determination of ET-1, NE, and MPO. Different lungs were homogenized (for protocol, see citrulline assay) for the immediate determination of NOS activities and for measurement of NOS gene expression and tissue MDA. The IR lungs were exposed to ischemia by stopping the perfusion and the ventilation for 90 min. The subsequent reperfusion lasted also 90 min after exchanging the complete perfusate.

The following drugs were administered during the entire IR cycle in combination with 5 nM human relaxin, which was the dose used in our previous work [3]:

- the non-selective nitric oxide synthase (NOS) inhibitor L-NAME (100 µM) (n = 8)
- the inducible NOS (iNOS) inhibitor 1400W (1 µM) (n = 6) [4]
- the neuronal NOS (nNOS) inhibitor S-methyl-L-thiocitrulline (l-SMTC) (10 µM) (n = 6) [5]
- the extracellular signal-regulated kinase-1/2 (ERK-1/2) inhibitor PD-98059 (50 µM) (n = 6) [6]
- the phosphatidylinositol-3 kinase (PI3K) inhibitor wortmannin (100 nM) (n = 6)
- the glucocorticoid receptor (GR) antagonist RU-486 (500 nM) (n = 3) [7]
- the endothelin type-B receptor (ETB) receptor antagonist A-192621 (500 nM) (n = 4) [8]
- the protein kinase A (PKA) inhibitors H-89 (5 µM) and KT-5720 (20 µM) (n = 3) [9].

In general, all drugs were also tested alone in the various settings (which is not always shown to assure clarity of figures) and, if not otherwise stated, were found to have no effect.

After IR experiments without any intervention (n = 6), lungs were homogenized as described below to test for the selectivity of the different NOS inhibitors. In another subset of lungs (n = 6 each), L-NAME, 1400W, SMTC, or relaxin were exclusively administered either during ischemia or during reperfusion.

In order to record the time course of NOS regulations and FKHRL1 phosphorylation lungs were also processed at baseline, after ischemia, or after reperfusion (n = 6 each).

Wet-to-dry weight ratio (W/D); NE, MPO, and ET-1in perfusate; as well as tissue MDA were determined as described in detail elsewhere [3].

### Citrulline Assay for NOS Activity

Lungs were homogenized over 30 min in ice-cold RIPA buffer (1% NP-40) (4 ml/mg tissue) containing 50 mmol/l NaF, 15 mmol/l Na_4_P_2_O_7,_ 2 mmol/l Na_3_VO_4_,10 µg/ml trypsin inhibitor, 1 mmol/l PMSF, 2 µg/ml aprotinin, 5 µg/ml leupeptin, 0.1 mmol/l okadaic acid, 5 mmol/l EDTA, and 1 mmol/l EGTA. The homogenates were centrifuged at 10.000 rpm at 4°C over 15 min; then, the supernatants were collected, and the protein concentration was determined using the Bradford method.

Enzyme reactions (final volume 200 µl) were carried out in the presence of 10 µl homogenization supernatant, 1 mmol/l NADPH, 15 µmol/l tetrahydrobiopterin, 1 µmol/l FAD, 1 µmol/l FAM, 1 mmol/l DTT, 10 µmol/l 3H-L-arginine, 1 mmol/l MgCl2, 100 nmol/l calmodulin, 300 µmol/l calcium, 0.2 mmol/l EDTA, and 0.2 mmol/l EGTA in HEPES buffer (50 mmol/l). Assays were also run in the presence of 1 mmol/l of the NOS inhibitor L-NG-nitro-L-arginine to detect non-specific production of 3H-citrulline as well as in the presence of 5 mmol/l EDTA to calculate calcium/calmodulin-independent NOS activity. Activity of constitutive NOS (eNOS plus nNOS) was then calculated by subtracting values for non-specific and for calcium/calmodulin-independent activity from the originally obtained values. In additional experiments, we also aimed at determining the contribution of nNOS which was regarded as the part of constitutive NOS sensitive to inhibition by SMTC (10 µmol/l).

In order to validate the selectivity of 1400W lung homogenates were pre-incubated over 30 min with 1400W prior to being diluted into the above-mentioned assays.

Assays were incubated for 60 min at 37°C; the reaction was stopped by adding ice-cold stop buffer (HEPES, pH 5.5, 10 mmol/l EDTA) and then transferred to columns containing 1 ml of Dowex 50w resin. After passage through the Dowex columns, 3H-citrulline was quantified in a scintillation counter.

### Quantification of Gene Expression of eNOS and iNOS

Total RNA was extracted from lungs using an RNeasy kit (Quiagen). The reverse transcriptase reaction contained 5 ng per µl total RNA, M-MLV reverse transcriptase (800 U), RNAseOUT (40 U), reverse primer (4 pM), dNTPs (0.5 mM), and supplied optimal buffers (all from Invitrogen). PCR was performed with 1 ng of cDNA template, 200 nM of iNOS or eNOS primers, and SYBR Green PCR master mix (Applied Biosystems). The eNOS (GeneBank accession: AJ011116) and iNOS primers (GeneBank Accession: D44591) were designed as described by Okada et al. [10]. The mRNA expression was standardised to the RPS11 (ribosomal protein S11) housekeeping gene. The expression of the target genes relative to the housekeeping gene was calculated as the difference between the threshold values for the two genes.

### Western Blotting

Lungs were homogenized over 30 min in ice-cold RIPA buffer (1% NP-40) (4 ml/mg tissue) containing 50 mmol/l NaF, 15 mmol/l Na_4_P_2_O_7,_ 2 mmol/l Na_3_VO_4_,10 µg/ml trypsin inhibitor, 1 mmol/l PMSF, 2 µg/ml aprotinin, 5 µg/ml leupeptin, 0.1 mmol/l okadaic acid, 5 mmol/l EDTA, and 1 mmol/l EGTA. The homogenates were centrifuged at 10.000 rpm at 4°C over 15 min; then, the supernatants were collected, and the protein concentration was determined using the Bradford method. After heating, equal amounts of protein (20 µg per lane) were separated by 8% SDS-PAGE and transferred to PVDF membranes (Hybond, Amersham). These membranes were blocked over 2 h at room temperature with RotiBlock (Roth).

Total FKHRL1 and phospho(Ser253)-FKHRL1 were detected with rabbit polyclonal IgG antibodies (sc-11351 and sc-12897, respectively) from Santa Cruz (dilution 1∶1000 and 1∶500, respectively). Horseradish peroxidase-conjugated antibodies (Santa Cruz) served as secondary antibodies. The signals were visualized by the ECL Plus chemoluminiscence system (abcam) and quantified using Quantity One (Bio-Rad Versadoc). For semi-quantitative analysis, densitometric data for phosphor-FKHRL1 were normalized to the total FKHRL1 protein.

### Cell Culture Experiments

Primary rat pulmonary artery endothelial cells (RPAEC) were prepared from rat pulmonary arteries by collagenase incubation, see [11]. Confluent RPAEC were detached with trypsin/EDTA and propagated in DMEM supplemented with 10% horse serum, 5% FCS, 100 µg/ml ECGS, 20 mmol/l HEPES, 2 mmol/l glutamin, 50 IU/ml penicillin, and 50 µg/ml streptomycin. Cells from passage 2 were used in these studies.

Primary rat pulmonary artery smooth muscle cells (RPASMC) were prepared according to Dahan et al. [12]. They were seeded in DMEM supplemented with 10% FCS, 50 IU/ml penicillin, and 50 µg/ml streptomycin. Cells were used 24 h after isolation.

Cells were transiently transfected with Silencer^R^ Select siRNA vectors targeting rat FKHRL1 or rat FOXB2 and scrambled siRNA using the Lipofectamine method according to the supplier’s instructions (Invitrogen). After 24 h of transfection, RPAEC and RPASMC were washed with DMEM supplemented with 4% horse serum plus 2% FCS and with 5% FCS, respectively, kept at rest for 8 h and then used for the following experiments: Cells were subjected to hypoxia (5% oxygen) for 90 min followed by another 90 min of normoxia. Control cells were kept under normoxia for 180 min. Concomitantly, cells were treated with vehicle, relaxin (5 nM), wortmannin (100 nM), PD-98059 (50 µM), and combinations thereof (n = 5 each).

The knock-downs were ascertained by 2 different antibodies against the N-terminus (sc-34897) and the C-terminus (sc-34894) of rat FKHRL1 and against rat FOXB2 (sc-132299) (Santa Cruz).

For NOS assays, cells were homogenized in RIPA buffer as described above for isolated lungs; 20 µl of extracts were then diluted into NOS assay buffer.

### Data Analysis

The data are presented as means ± S.E.M. Differences between groups were analysed using the Kruskal-Wallis ANOVA on ranks or, in the case of two independent variables (time and group), a non-parametric two-way ANOVA. After global testing, individual groups were compared using the Mann-Whitney rank sum test with Bonferroni-Holm adjustement of p. An error probability of p<0.05 was regarded as significant.

## Results

### Relaxin’s Beneficial Action is Completely NO-dependent

As recently shown [3] relaxin beneficially affected all chosen parameters of IR injury. The non-specific NOS inhibitor L-NAME completely abolished relaxin’s protective effect (not shown). Given alone during the complete IR experiment L-NAME changed none of the readouts (data not shown).

In contrast, the selective nNOS inhibitor, SMTC; the 2 different PKA inhibitors, KT-5720 and H-89; the ETB antagonist, A-192621; and the GR antagonist, RU-486, did not show any effect on relaxin’s beneficial action during IR (data not shown).

### Validation of the Selectivity of iNOS Inhibition by 1400W

**Figure 1:**
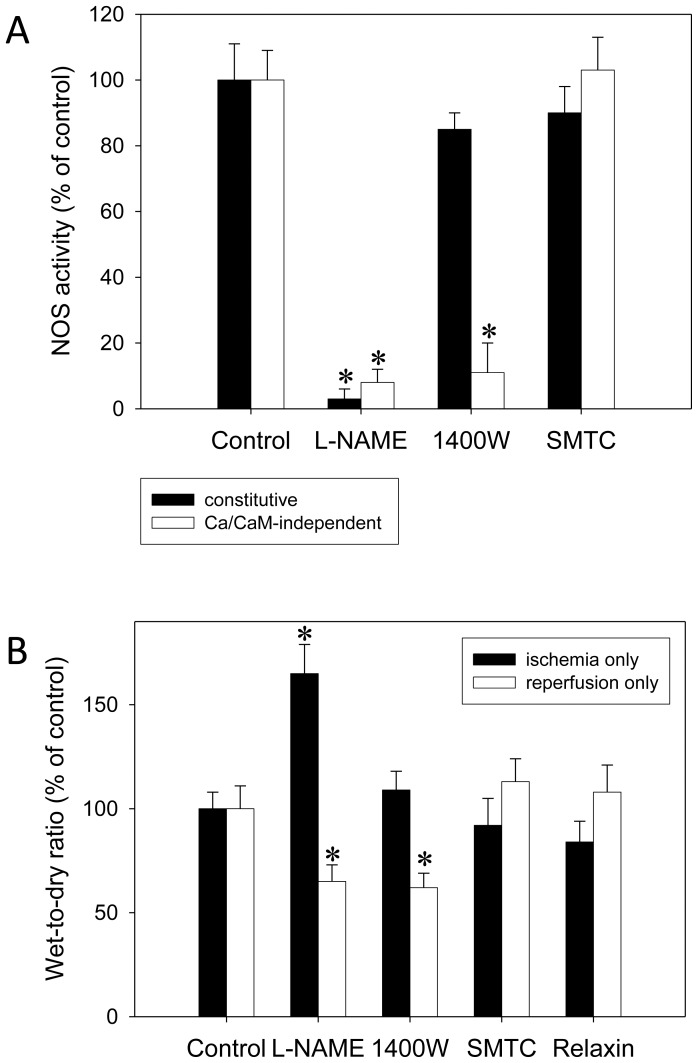
Validation of the selectivity of iNOS inhibition by 1400W. Isolated rat lungs were perfused with buffer in recirculatory mode and underwent an IR cycle comprised of 90(A) NOS assays conducted in lung homogenates after IR experiments without intervention (n = 6 each) showing that 1400W inhibited iNOS (Ca/CaM-independent activity) with sufficient selectivity over constitutive NOS. L-NAME, a non-selective NOS inhibitor, was given at 100 µM, 1400W, a selective iNOS inhibitor, at 1 µM, and SMTC, an nNOS inhibitor, at 10 µM. (B) In another subset of lungs (n = 6 each), L-NAME, 1400W, SMTC, or relaxin (5 nM) were exclusively administered either during ischemia or during reperfusion. Here, the wet-to-dry ratio is shown which was representative for all other readouts. While L-NAME affected both ischemia and reperfusion 1400W impacted only on reperfusion; relaxin (when not applied during the full IR cycle) had no effect. There was no relevant effect of nNOS inhibition at all. P<0.05; *vs. control.

In NOS assays conducted after IR experiments without intervention (Figure 1A), 1400W inhibited constitutive NOS (eNOS and nNOS) only slightly, by 15±5% (NS), but iNOS significantly, by 89±9%. L-NAME completely inhibited all NOS forms, and SMTC showed no significant effect.

In other experiments (Figure 1B), L-NAME, 1400W, SMTC, or relaxin were exclusively administered either during ischemia or during reperfusion. L-NAME was harmful when given in ischemia but improved W/D ratio when restricted to reperfusion. In contrast, 1400W was neutral in ischemia but protective in reperfusion. SMTC had no effect at all. Relaxin, in contrast to its effects when administered during the full IR cycle, did not affect IR injury if applied either during ischemia or during reperfusion. This held true for all readouts (W/D ratio, NE, MPO, ET-1, MDA, and MPAP) while only the W/D ratio is shown in Figure1.

### Selective iNOS Inhibition by 1400W Completely Prevents Relaxin-related Protection

**Figure 2:**
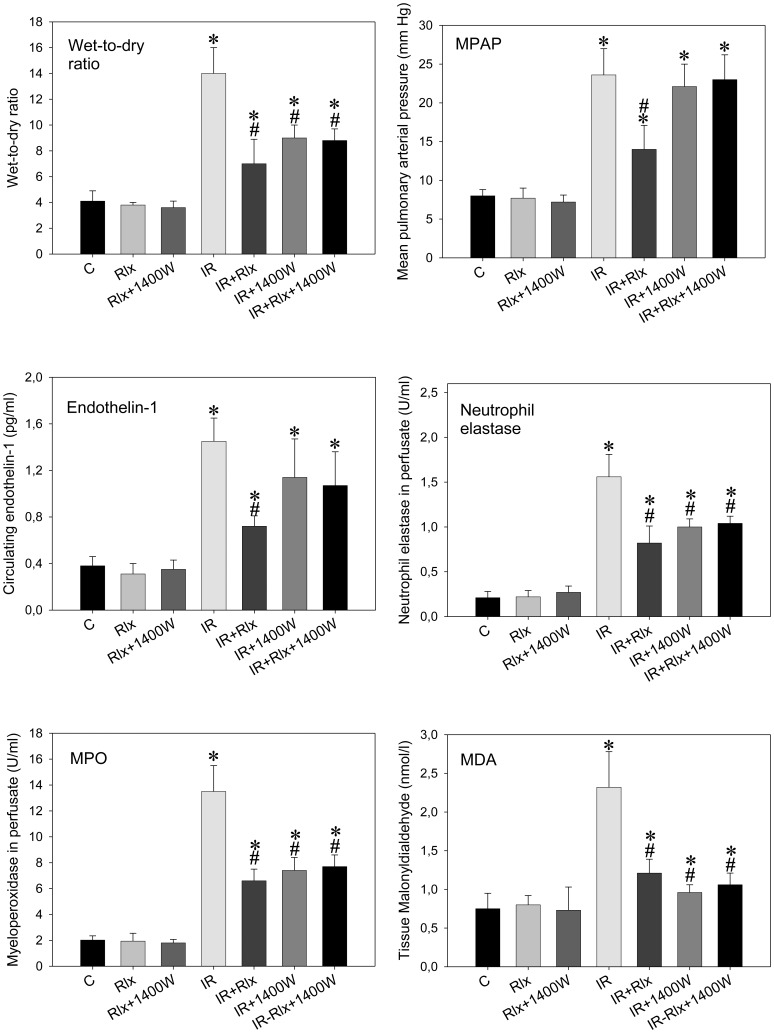
Selective iNOS inhibition by 1400W prevents relaxin-related protection. Wet-to-dry weight ratio, mean pulmonary arterial pressure (MPAP), neutrophil elastase, myeloperoxidase (MPO), and endothelin-1 in perfusate, as well as tissue malonyldialdehyde (MDA) in isolated rat lungs perfused with buffer in recirculatory mode and subjected to 90 min ischemia followed by 90 min reperfusion. Experiments (n = 6 each) were carried out in the presence of vehicle (control), 5 nM of relaxin (Rlx), the selective iNOS inhibitor 1400W (1 µ), and combinations thereof. Both 1400W (IR+1400W) and relaxin (IR+Rlx) exerted beneficial effects; when applied in the presence of 1400W relaxin did not add protection (IR+Rlx+1400W). P<0.05; *vs. control; #vs. IR.

Figure 2 summarizes all parameters of IR injury in isolated rat lungs: IR caused pulmonary edema (i. e., increased W/D ratio), pulmonary hypertension, as well as increased levels of ET-1, NE, MPO, and MDA. Relaxin clearly mitigated this IR injury in all its facets and blunted the increase from controls to IR in each of the different readouts by more than 50%. Selective inhibition of iNOS by 1400W alone also resulted in protection of the lungs: the drug beneficially influenced all readouts but pulmonary hypertension and ET-1. In combination, however, 1400W inhibited the effects of relaxin on IR injury completely; that is, in none of the readouts, relaxin added any protection against IR damage in the presence of 1400W.

### Both ERK-1/2 and PI3K Inhibition Abolish Relaxin’s Protective Effect

**Figure 3:**
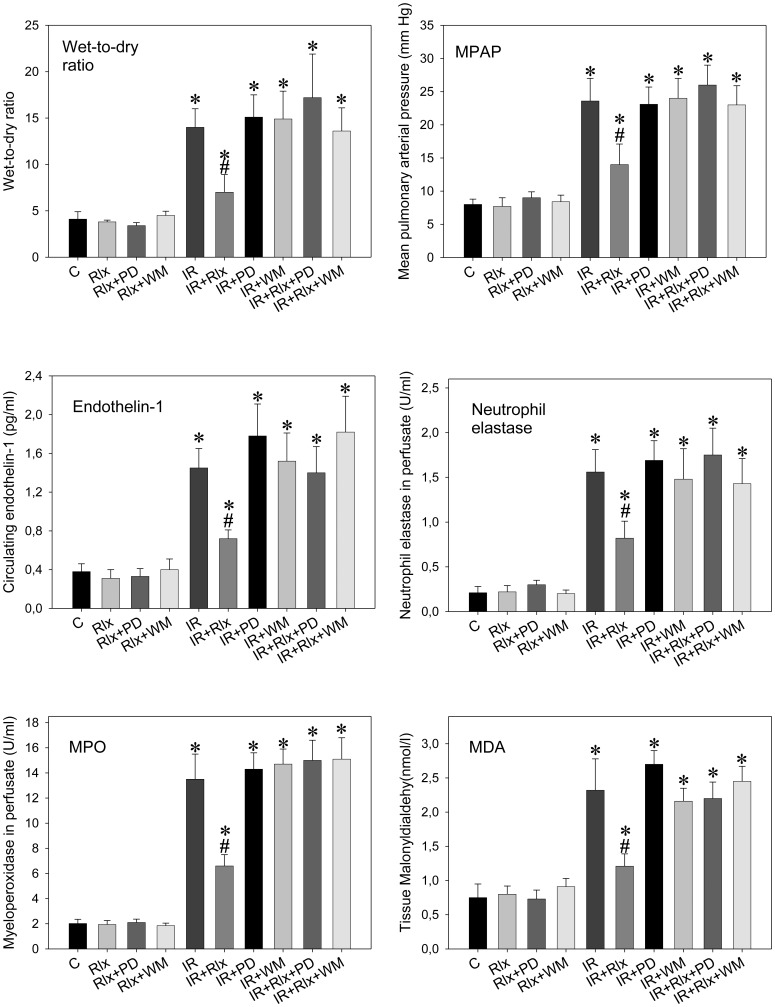
Both ERK-1/2 and PI3K inhibition abolishes relaxin’s protective effect. Wet-to-dry weight ratio, mean pulmonary arterial pressure (MPAP), neutrophil elastase, myeloperoxidase (MPO), and endothelin-1 in perfusate, as well as tissue malonyldialdehyde (MDA) in isolated rat lungs perfused with buffer in recirculatory mode and subjected to 90 min ischemia followed by 90 min reperfusion. Experiments (n = 6 each) were carried out in the presence of vehicle (control), 5 nM of relaxin (Rlx), the ERK-1/2 inhibitor PD-98059 (PD) (50 µmol/l), the PI3K inhibitor wortmannin (WM) (100 nM), and combinations thereof. Inhibition of either ERK-1/2 or PI3K did not change IR (IR+PD and IR+WM) but completely prevented the effects of relaxin therein (IR+Rlx+PD and IR+Rlx+WM). P<0.05; *vs. control; #vs. IR.

Figure 3 shows the complete dependence of the relaxin effect of both ERK-1/2 and PI3K activation: the ERK inhibitor, PD-98059, and the PI3K inhibitor, wortmannin, had no relevant effects when given alone but abolished all relaxin-related actions.

### Relaxin-related Stimulation of iNOS is Mediated via ERK-1/2 and Counter-balanced by PI3K

**Figure 4:**
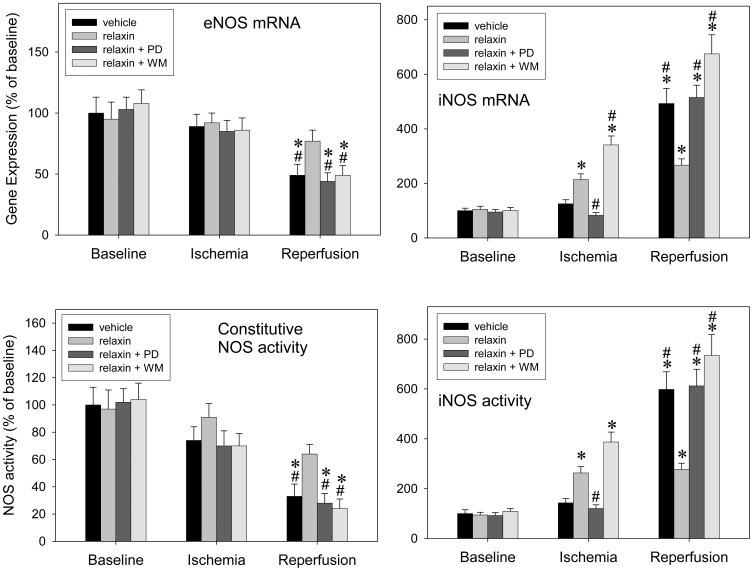
eNOS-iNOS cross-talk: relaxin-related stimulation of iNOS is mediated via ERK-1/2 and counter-balanced by PI3K. Isolated rat lungs perfused with buffer in recirculatory mode were processed at baseline, after 90(n = 6 each) in order to determine gene expression (upper panel) and activity (lower panel) of eNOS and iNOS. Experiments were carried out in the presence of vehicle (control), 5 nM of relaxin, the ERK-1/2 inhibitor PD-98059 (PD) (50 µmol/l), the PI3K inhibitor wortmannin (WM) (100 nM), and combinations thereof. The natural course of IR, down-regulation of eNOS and up-regulation of iNOS in reperfusion, is altered by relaxin, into eNOS maintenance in reperfusion and early moderate iNOS induction in ischemia. Relaxin’s action is suppressed both by ERK-1/2 and PI3K inhibition. P<0.05; *vs. baseline; #vs. relaxin.

Figure 4 shows gene expression and activity of eNOS and iNOS determined at baseline, after ischemia, and after complete IR: The natural course of IR injury (vehicle group) was characterized by concomitant down-regulation of eNOS expression and activity at the end of the reperfusion phase, to approximately 50% of baseline values. In parallel, iNOS was induced during reperfusion, to approximately 500% of baseline levels. Relaxin prevented the IR-induced marked down-regulation of eNOS while leading to an early iNOS up-regulation in ischemia. iNOS remained elevated but below the levels seen in the vehicle group during reperfusion. ERK-1/2 inhibition prevented the relaxin-related early iNOS up-regulation which, in turn, was aggravated by PI3K inhibition. However, both interventions abolished the relaxin effect on eNOS and the relaxin-related moderate level of iNOS induction during reperfusion. ERK-1/2 or PI3K inhibition alone (without relaxin) did not change the course of the experiment (not shown in figure 4).

The use of a two-way ANOVA allowed for interaction testing. Whereas no significant overall interaction “time×group” was found for eNOS mRNA it was detectable for iNOS mRNA and activity (p for overall testing <0.00001 either). Then, a significant interaction between relaxin and wortmannin towards iNOS could be inferred from the finding that upon pairwise testing, “relaxin+wortmannin” was different from both “relaxin alone” (p<0.00001) and “wortmannin alone” (p = 0.0004) while there was no difference between the two latter ones (p = 0.29, relaxin vs. wortmannin). Such an interaction was not determined for relaxin and the ERK inhibitor, PD-98059.

### Relaxin-PI3K- phosphoFKHRL1 Signalling is Essential to iNOS Inhibition

**Figure 5:**
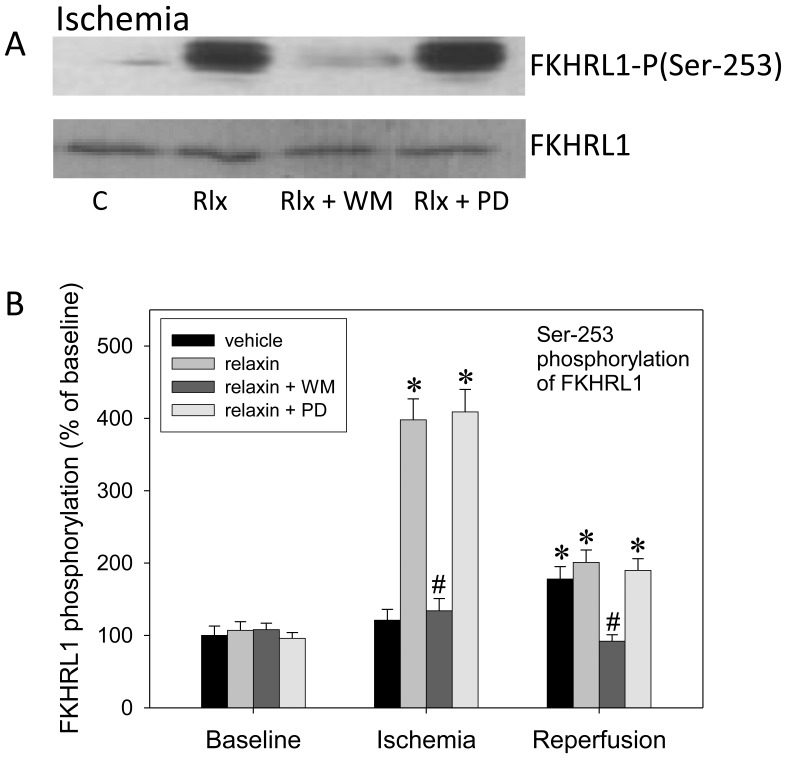
Relaxin, via PI3K, phosphorylates FKHRL-1. Isolated rat lungs perfused with buffer in recirculatory mode were processed at baseline, after 90(n = 6 each) in order to determine protein expression (panel A) and Ser-253 phosphorylation (panel B) of the forkhead transcription factor, FKHRL1. Experiments were carried out in the presence of vehicle (control), 5 nM of relaxin, the ERK-1/2 inhibitor PD-98059 (PD) (50 µmol/l), the PI3K inhibitor wortmannin (WM) (100 nM), and combinations thereof. (A) Representative Western blot from lung homogenates produced after ischemia. (B) Quantification of Western blot data (n = 6 per group). Relaxin causes FKHRL-1 phosphorylation during ischemia in a PI3K (wortmannin)-dependent fashion; there is no dependence of ERK-1/2. P<0.05; *vs. baseline; #vs. relaxin.

Since PI3K-related phosphorylation of the forkhead transcription factor, FKHRL1, has been shown to inhibit iNOS induction in lung cells in inflammatory states [13] we determined total and phosphorylated FKHRL1 in lung homogenates at baseline, after ischemia, and after the full IR cycle (Figure 5). Relaxin, in a PI3K-dependent fashion, markedly elevated phospho-FKHRL1 in ischemia compared with vehicle, to approximately 400% of baseline levels. ERK-1/2 inhibition did not influence this action. PD-98059 or wortmannin had no effect on FKHRL1 when given alone (not shown). The same effect of relaxin on FKHRL1 phosphorylation could also be shown in RPAEC and RPASMC (not shown).

**Figure 6:**
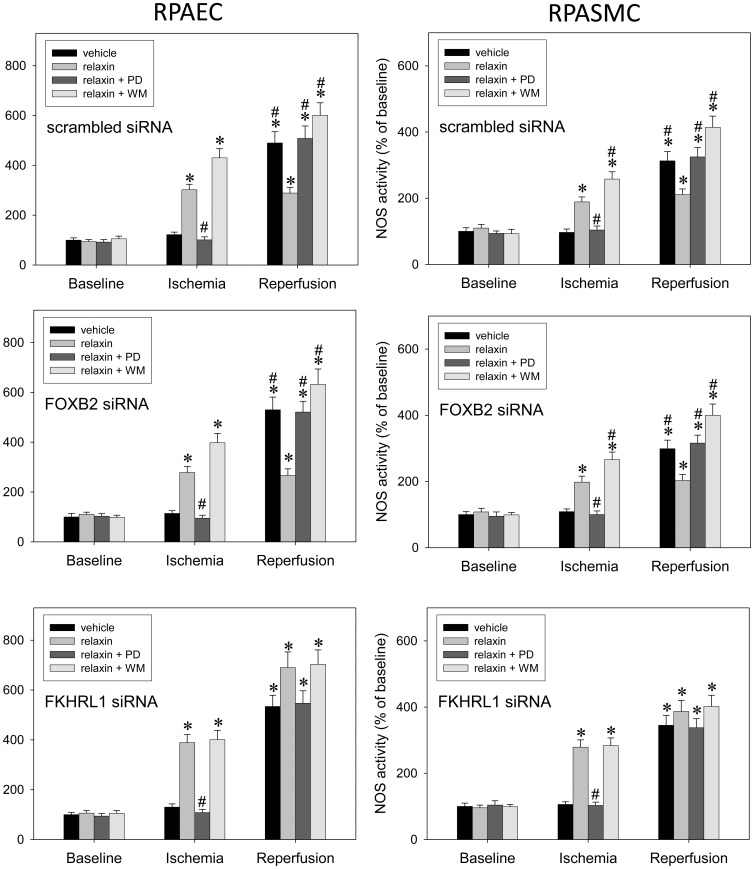
FKHRL1 is essential for relaxin signaling towards iNOS. Primary rat pulmonary artery endothelial cells (RPAEC, left side) and rat pulmonary artery smooth muscle cells (RPASMC, right side) were processed at baseline, after 90 min hypoxia (5% oxygen), or after another 90 min of normoxia (n = 5 each) in order to determine iNOS activity. Experiments were carried out in the presence of vehicle (control), 5 nM of relaxin, the ERK-1/2 inhibitor PD-98059 (PD) (50 µmol/l), the PI3K inhibitor wortmannin (WM) (100 nM), and combinations thereof. Prior to experiments, cells had been transfected with scrambled siRNA (control), FOXB2 siRNA, or FKHRL1 siRNA. While both transfection with scrambled siRNA and knock-down of non-related forkhead factor, FOXB2, had no influence knock-down of FKHRL-1 abolished the susceptibility of relaxin’s effect towards PI3K inhibition in ischemia and equalized the extent of iNOS induction in reperfusion. P<0.05; *vs. baseline; #vs. relaxin.

Using a similar protocol in RPAEC and RPASMC (except for the fact that IR was mimicked here as hypoxia) (Figure 6), we confirmed that FKHRL1 phosphorylation was critical to the PI3K-dependent inhibitory action of relaxin towards iNOS: Transfection with scrambled control siRNA or siRNA targeting a different forkhead transcription factor, FOXB2, affected neither the sensitivity of the relaxin effect to wortmannin nor the relaxin-related pattern of iNOS induction. In contrast, knock-down of FKHRL1 prevented both the wortmannin-related increase in NOS activity as compared to relaxin administration alone and the relaxin-related moderate level of iNOS up-regulation during reperfusion as compared with all other groups.

Corresponding to the data in figure 4, there was a significant statistical interaction between relaxin and wortmannin, detectable after transfection with scrambled or FOXB2 siRNA but abolished upon FKHRL1 knock-down.

In control cells kept under constant normoxia for 180 min, there was no relevant regulation of iNOS (data not shown).

## Discussion

The rationale behind studying the effects of relaxin in lung IR is given by its broad spectrum of anti-inflammatory, vasodilatory, and endothelium-protecting properties [3], [11], [14]–[16] and by reports on its protective action in myocardial IR [17]–[19]. The present study investigated possible mechanisms by which relaxin may exert its protective effects in IR-induced injury in rat lungs. The main findings of the present work are as follows: 1) Relaxin mitigated IR-induced lung injury via *early* and *moderate* iNOS induction. 2) This effect critically depended on a subtle balance between ERK-1/2 activation, which promoted iNOS expression, and counter-regulatory PI3K activation. 3) The relaxin-PI3K signaling was critically dependent on the phosphorylation of forkhead transcription factor, FKHRL1.

In general, relaxin has been found to target a variety of pathways via its receptor, RXFP1, including cAMP/PKA, NO via all different NOS enzymes, ETB receptors, ERK-1/2, and PI3K [16]. In addition, it has also been shown to bind to and activate GR [11], [20]. We found here that non-specific inhibition of NO synthase with L-NAME abolished the protective effect of relaxin in IR-induced lung injury. It is well known that in the pulmonary vasculature, NO is normally produced in endothelial cells from eNOS, is involved in the regulation of vascular tone, counter-balances leukocyte and platelet activation, and maintains normal endothelial permeability [21]. Similarly, in animals and humans, NOS inhibition induced an increase in pulmonary vascular resistance [22].

Under pathological conditions such as pulmonary IR, the increased vascular tone may be partially explained by an attenuation of NO synthesis from eNOS and/or decreased NO bioactivity [2]. During IR, iNOS is markedly induced at the transcriptional level [21] via activation of the transcription factor NF-kβ [23]. This causes synthesis of excessive amounts of iNOS-derived NO. In this setting, NO is no longer a mediator of vascular homeostasis and potentiates injury by forming peroxynitrite in a reaction with the likewise increased superoxide, which acts as toxic vasoconstrictor generating hydroxyl radical [24]. Peroxynitrite formation decreases the bioavailability of NO and participates in a host of structural alterations due to its ability to initiate lipid peroxidation [25]. Peroxynitrite also oxidizes the essential eNOS cofactor tetrahydrobiopterin leading to eNOS uncoupling with further superoxide generation [25]. Moreover, increased production of peroxynitrite inhibits the catalytic centre of PI3K, with two major implications: first, since PI3K-Akt activation is important to eNOS activation generation of NO from eNOS is diminished; and second, since PI3K-mediated phosphorylation of the forkhead transcription factor FKHRL1 dampens iNOS induction iNOS-related NO production is further enhanced [2], [13].

Studies on vascular endothelial and smooth muscle cells have clearly demonstrated that relaxin can promote vascular NO generation, either by activating eNOS or by increasing, in a cell type-dependent manner, the expression of the three different NOS isoforms [16]–[18]. In line with this, Masini demonstrated that the inhibition by relaxin of neutrophil activation depended on rapid (within 60–90 min) and moderate (2–3fold) iNOS induction [18].

The current study demonstrates that the mitigation of IR-induced lung injury by relaxin is promoted by an early and moderate induction of iNOS. This effect mainly consists of an up-regulation of iNOS mRNA and Ca/calmodulin-independent NO production during *ischemia*. In the reperfusion period, mRNA and NO production remain constantly elevated but are significantly reduced compared with vehicle. In addition, relaxin given only during reperfusion had no effect.

The conclusion of iNOS being prevailingly responsible for relaxin’s effect herein is based on the following findings: Both non-specific NOS inhibition by L-NAME and specific iNOS inhibition by 1400W completely suppressed the relaxin-related protection; the nNOS-specific inhibitor SMTC did not show any effect; and modulating the course of relaxin-mediated iNOS induction by parallel ERK-1/2 or PI3K inhibition also abolished relaxin’s beneficial action.

Our findings (figures 1 and 4) regarding the time course of NOS induction and the efficacy of 1400W confirmed the deleterious involvement of iNOS in the course of lung IR. iNOS inhibition alone by 1400W clearly attenuated IR damage which can be concluded from all parameters depicted in figure 2 with the exceptions of MPAP (neutral effect) and ET-1 (trend towards attenuation). How then could the very same iNOS inhibition prevent the effect of relaxin which itself was similarly beneficial as confirmed by all recorded parameters in figure 2? In our opinion, relaxin modified the natural course of IR by inducing iNOS earlier but moderately, and this appears to be protective [18], [26]. Once given together with 1400W relaxin did not add any protective effect (compare IR+1400W versus IR+1400W+relaxin) no matter which parameter of figure 2 is considered. The fact that for MPAP and ET-1, the group “IR+relaxin” appears to differ from “IR+relaxin+1400W” and also from “IR+1400W” merely reflects the distinction between complete inhibition by 1400W of markedly up-regulated iNOS (i. e., inhibition of the natural course of IR) and earlier but moderate iNOS induction by relaxin (i. e., therapeutic modification in the presence of relaxin).

The selectivity of 1400W for iNOS over eNOS/nNOS was validated in our experimental setting: First, 1400W proved sufficiently selective in NOS assays from lung homogenates. Second, and in contrast to L-NAME, it affected only the reperfusion events when iNOS was evidently induced but was neutral during ischemia when eNOS function was still normal.

Moreover, relaxin seems to influence what is called eNOS-iNOS cross-talk, a kind of reciprocal regulation involving iNOS stimulation at the cost of eNOS expression and activity [27]. In the presence of relaxin, eNOS expression was significantly increased compared to the vehicle group during reperfusion. The NOS cross-talk may be explained differently: First, low levels of NO production from eNOS inhibit stimulation of iNOS transcription by inactivating NF-κβ [28], stabilizing the NF-κβ inhibitory factor, Iκβ, or directly interfering with binding of NF-κβ by nitrosylating the p50 subunit of NF-κβ [2]. IR may increase ROS production thereby decreasing NO availability and expediting iNOS expression. Early and moderate iNOS induction by relaxin may act to prevent IR-related cell activation and the concomitant respiratory burst (compare our findings regarding NE and MPO) which then maintains NO bioavailability. Alternatively, relaxin is known to attenuate the stimulated release of TNF-α [20], a key cytokine in IR injury [2], [29]. TNF, in turn, is an inducer of the described eNOS-iNOS shift [29]. Whether this applies to the model used here remains to be investigated.

Furthermore, the mechanism of action of relaxin towards iNOS seems to be mediated by parallel activation of the ERK-1/2 and PI3K cascades. Causal implication of these cascades was confirmed by the finding that both PD-98059 and wortmannin completely suppressed the protective relaxin effect. Whereas ERK-1/2 inhibition prevented the early iNOS up-regulation during ischemia PI3K inhibition by wortmannin enhanced this effect. Accordingly, relaxin-related ERK-1/2 activation, which is known to target NF-κB [14], [16], stimulates iNOS expression and is counter-balanced by PI3K, another established relaxin pathway [11], [16], via phosphorylation and suppression of FKHRL1. The latter mechanism, PI3K-mediated FKHRL1 phosphorylation, has been found to inhibit iNOS induction in lung cells [13], and we could show relaxin-related FHRL1-phosphorylation in our lung model (Figure 5). Moreover, in pulmonary endothelial and smooth muscle cells (Figure 6), FKHRL1 was confirmed to be critical to the described relaxin-PI3K signaling. In concert, ERK-1/2 and PI3K activations act to promote a moderate NO rise in early ischemia sufficient to keep the subsequent inflammatory events at bay.

With regard to iNOS induction in IR, relaxin and PI3K inhibition by wortmannin enhance each other. An interaction was identified by statistical analysis and supports the fact that PI3K activation represents an essential part of relaxin’s signaling. This interaction should be considered in the design of relaxin-based therapies since enhanced iNOS induction may be harmful.

**Figure 7:**
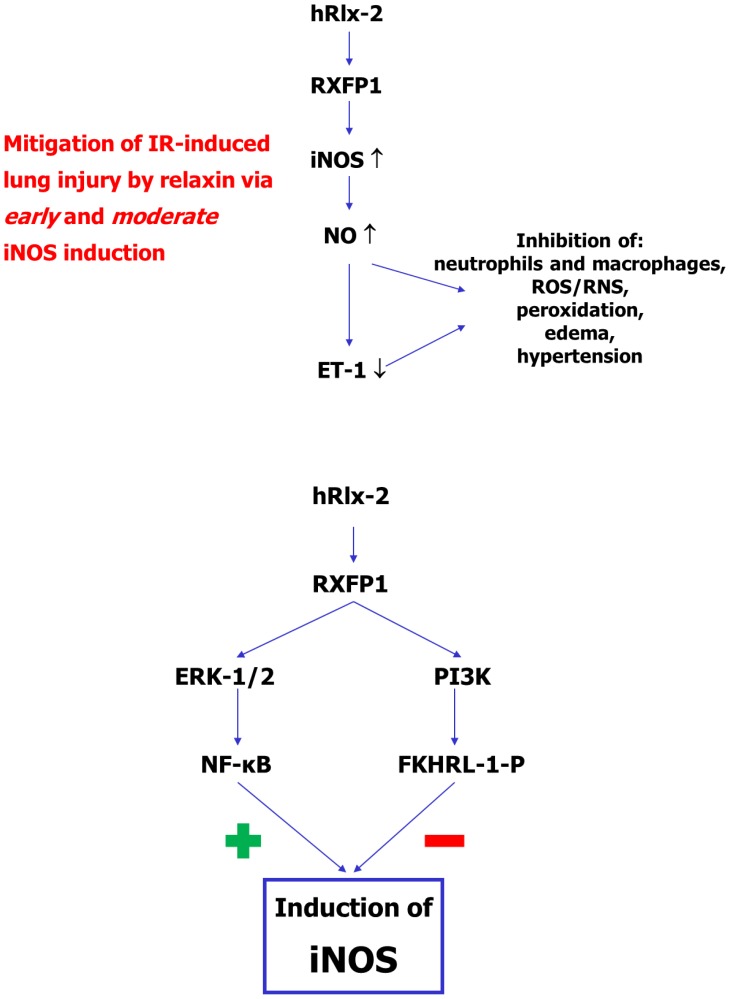
Summary of the proposed relaxin signaling during lung IR. Upper panel: In a short-term IR cycle in rat isolated lungs, relaxin induced iNOS in a manner (early in ischemia but moderately in level) distinct from the natural course (later but marked in reperfusion). This moderately elevated NO generation suppresses the IR-related ET-1 surge, both effects act in concert to inhibit cell activation and subsequent tissue damage. Lower panel: Early and moderate iNOS induction by relaxin is dependent on a subtle balance between stimulatory ERK-1/2-NF-κB and inhibitory PI3K-FKHRL-1 pathways.

Endothelin-1, which acts as vasoconstrictory, permeability-increasing, and pro-inflammatory mediator [30], is likewise increased by IR in our experimental model and NO-dependently blunted by relaxin. NO, which lowers intracellular calcium levels, is an established negative transcriptional regulator of ET-1 [30]. In this short-time model, another effect of NO, namely the inhibition of the release of mature ET-1 from endothelial Weibel-Palade bodies [31], is likely to come into play. This makes the protective effect of relaxin in this model attributable to favorable modulation of at least two key players in IR, NO and ET-1, as summarized in Figure 7.

As to the negative results of pharmacological interventions, relaxin has been demonstrated to stimulate endothelial and epithelial ETB expression via a Ras-independent Raf-1–MEK-1–ERK-1/2 pathway that activates NF-κβ [14]. Alternatively, relaxin activates matrix metalloproteases (MMP-9, MMP-2) which generate the longer and ETB-selective ET-1(1–32) from big ET-1 [15]. Either effect renders relaxin a functional endothelin-1 antagonist. In addition, relaxin has been demonstrated to act as GR agonist and to inhibit stimulated release of pro-inflammatory cytokines, in particular TNF-α [20]. As opposed to that, in this short-time setting, the involvement of ETB and GR did not play any significant role. However, the ETB and GR pathways may be relevant to longer-term effects of relaxin in IR models.

At last, relaxin-mediated ERK-1/2 activation has been reported to occur downstream of cAMP/PKA but PKA-independent effects on ERK have also been established depending on the cell type investigated [16]. Our findings in isolated lungs, for which data are scarce, showed PKA-independent ERK targeting by relaxin, presumably via Gβγi3 [16].

As to the limitations to the present study, we investigated IR effects in a short-time setting, which may not be accurately comparable to real clinical IR states. However, isolated lungs exhibit key characteristics of IR-related pulmonary alteration, in particular edema, pulmonary hypertension, cell activation, and ROS generation [32].

Finally, lungs were perfused with saline buffer, which excludes many cell-mediated events that may affect pulmonary perfusion pressure and vascular permeability. Blood-free perfusion, on the other hand, does not affect the number of neutrophils adhering to the endothelium of isolated lungs [32] but it inevitably prevents recruitment exceeding the number of these already adhering cells. This fact may cause an underestimation of the neutrophil-related damage, particularly of the levels of neutrophil elastase and myeloperoxidase, and, consequently, of relaxin’s protective effects.

In conclusion, in this short-time experimental setting, human relaxin-2 exerts its protective effect on IR-induced lung injury via early and moderate iNOS induction, dependent on balanced ERK-1/2 and PI3K stimulation (as summarized in Figure 7).
